# Effects of Nursing Interventions During Delivery on Pain Relief and Labor Progression in Vaginal Deliveries

**DOI:** 10.7759/cureus.84132

**Published:** 2025-05-14

**Authors:** Qi Luo, Zhaie Lu, Binbin Xu, Qirong Feng

**Affiliations:** 1 Obstetrics: Midwifery, Women and Children's Hospital of Ningbo University, Ningbo, CHN; 2 Obstetrics: Delivery Room, Women and Children’s Hospital of Ningbo University, Ningbo, CHN; 3 Gynecologic Oncology, Ningbo Women and Children's Hospital, Ningbo, CHN; 4 Obstetrics Nursing: Maternity Ward, Women and Children’s Hospital of Ningbo University, Ningbo, CHN

**Keywords:** delivery women, labor progression, nursing intervention, pain, vaginal delivery

## Abstract

Background

The beneficial influence of nursing interventions upon the mother during natural childbirth is profound, though it has, as yet, not been tangibly identified. This is particularly important and of clinical relevance in the regulation of pain levels experienced by the mother in such an important life event.

Aims

This study aims to evaluate the effects of nursing intervention during delivery on pain relief and labor progression in vaginal delivery women.

Methods

A total of 86 vaginal delivery women from July 2023 to August 2024 were included and divided into two groups through a table of random numbers: Intervention group (n=43, receiving enhanced nursing intervention during delivery) and a Control group (n=43, receiving routine nursing intervention). The pain during delivery, duration of labor, childbirth attitudes questionnaire (CAQ), self-rating anxiety scale (SAS), and delivery outcomes were evaluated between the groups.

Results

Compared with the Control group, the visual analog scale (VAS) scores of the Intervention group at different time points (latent phase, acceleration phase, and deceleration phase) were lower, and the average time spent on the first, second, and third stages of labor was shorter. The scores of CAQ, SAS, and the usage rate of oxytocin were lower. In addition, there was less bleeding after two hours postpartum in the Intervention group (*P*<0.05). There was no difference in Apgar scores at five minutes of birth for newborns between the two groups (*P*>0.05).

Conclusion

Strengthening nursing interventions during vaginal delivery for vaginal delivery women can alleviate delivery pain, promote labor progression, alleviate childbirth fear and anxiety, and improve delivery outcomes.

## Introduction

Vaginal delivery is a common and ideal method of childbirth, which is beneficial for maternal and child health. However, the stimulation generated by the dilation of the cervix, disappearance of the cervical canal, and continuous contraction of the uterus during delivery can gradually increase the pain of childbirth, and even reach unbearable visceral pain at the highest level (Level 12) [1,2]. For primiparous women who have not experienced childbirth, the lack of correct understanding of the delivery process, combined with the decreased levels of progesterone and increased sensitivity in late pregnancy, can make it difficult for them to tolerate the pain of childbirth, leading to a bias in choosing the delivery method, resulting in a high cesarean section rate, negative delivery experience, and reduced quality of postpartum life. Furthermore, the beneficial influence of nursing interventions upon the mother during natural childbirth is profound, though it has, as yet, not been tangibly identified. This is particularly important and of clinical relevance in the regulation of pain levels experienced by the mother in such an important life event.

Therefore, it is necessary to continuously improve the nursing mode of vaginal delivery assistance to enhance the confidence of vaginal delivery mothers in childbirth, improve their ability to cope with delivery pain, reduce their perception of pain during labor, alleviate fear-tension-pain syndrome, promote smooth progress of labor, and meet higher-level physical and mental needs [3-5]. Nursing intervention during delivery can optimize the delivery experience and ensure the safety of mothers and infants by providing specialized and personalized support measures. However, its practicality for vaginal delivery women still needs further confirmation.

Aims

This interventional study selected 86 vaginal delivery women (from July 2023 to August 2024), to explore the effects of detailed nursing intervention during delivery, together with the potential influence of such detailed nursing interventions upon pain relief and labor progression in such women. The aims of this study are multifaceted and focus on evaluating the impact of enhanced nursing interventions during childbirth. Specifically, the study seeks to assess the effects of such interventions on the intensity of delivery pain and the duration of labor. Additionally, it aims to examine how enhanced nursing care influences maternal attitudes toward childbirth and levels of anxiety. Lastly, the study analyzes the outcomes of delivery, including the use of oxytocin and the incidence of postpartum bleeding, in relation to the implementation of enhanced nursing support.

## Materials and methods

Patient selection

This investigation involved study participants presenting at the Ningbo Women and Children's Hospital, Ningbo city, Zhejiang province, China. This study received approval from the Ethics Committee of Ningbo Women and Children's Hospital, with Approval No. EC2020-KY-081.

Primiparous women who underwent natural contractions, entered labor, had an unopened cervix, and had a Bishop score greater than 7 points were included in the study. A total of 86 vaginal delivery women from July 2023 to August 2024 were included and divided into two groups through a table of random numbers: an Intervention group (n=43) and a Control group (n=43). The results of patient profiling following such a selection process are highlighted in the Results section.

The inclusion criteria were: First delivery, full-term pregnancy; Single pregnancy, with normal fetal development; No indications related to cesarean section, suitable for vaginal delivery; Accepted research methods and signed informed consent form.

The exclusion criteria were: Fetal distress or macrosomia; Those who were unable to undergo vaginal delivery due to complications or comorbidities during pregnancy; Patients with severe organic lesions such as liver, kidneys; Individuals with mental and intellectual issues; Patients who transitioned from childbirth to cesarean section; Individuals with pelvic abnormalities.

Experimental protocol

Concerning the Intervention group (strengthening nursing intervention during delivery), patients were prepared before delivery, according to the following steps.

Health Education

Delivery room nurses conducted a comprehensive examination and evaluation of the condition of the mother and fetus, focusing on introducing the vaginal delivery process, signs of labor (regular contractions, redness, and water breakthrough), methods for judging regular contractions (interval of 5-6 minutes per time), identification of effective contractions (regular contractions lasting >30 seconds), self-monitoring of fetal movement, characteristics and mechanisms of pain at different time points, methods and significance of pain relief nursing, self-emotion regulation skills, and preparation of materials for the mother and newborn to the mother and their families.

Physiological Care

The first step for the delivery room nurses was to inform the mother of methods and key points for relieving pain such as massage relaxation, shifting attention, and respiratory control. The second step was to guide the mother to maintain a free position. The third step was to advise the mother to have multiple meals with small amounts of each high-calorie and easily digestible food. The fourth step was to advise the mother to avoid significantly increasing abdominal pressure and vigorous exercise.

Psychological Nursing

Nursing staff should not be harsh on women in delivery, maintain a warm attitude, actively communicate, patiently listen to their concerns and needs, and provide personalized guidance. 

Environmental Care

Keep the ward quiet and undisturbed.

Concerning care during childbirth, the following steps were implemented.

 *Pain Relief Care*

The midwife gently massaged the abdominal area and buttock muscles below the iliac wings of the delivery woman, played soothing music, maintained a free position, and guided the delivery woman to close her eyes and imagine walking on the beach to relieve the pain of childbirth.

Physiological Nursing

First, the midwife guided the parturient to maintain contour breathing when the cervix was opened by 1-6 cm, shallowed breathing and contoured breathing when the cervix was opened to 6-10 cm, and abdominal breathing during the second stage of labor. Second, the midwife guided the parturient to empty her bladder regularly, remain engaged in indoor activities, avoid drinking functional drinks, and take care of herself to prevent falls, including bed-falls. Third, the midwife assisted the delivering mother in adopting the correct delivery posture and techniques, helping the fetus to be delivered smoothly. For example, for those in the posterior position of the pillow, the delivery ball can be used to promote delivery, and time interval relaxation training can be combined after the cervix was opened to 2 cm (the midwife informed the mother to relax the whole body, open the eyes and mouth 3-4 times, massaged the neck, pelvis, chest, and abdomen, and cooperated with abdominal breathing when contractions occur, as well as allowing the mother to count the number of breaths). At the same time, during the contractions, the mother was given 3 mL of a sweet drink orally. Fourth, midwives and delivery room nurses jointly monitored the fetal heart rate, fetal position, and other conditions. Fifthly, after the fetus was delivered, they closely observed the uterine contractions and bleeding volume of the mother to ensure postpartum safety.

Psychological Care

Midwives should promptly inform the delivery women about the condition of the fetus and the progress of the labor process, respond to delivery women in a timely manner, encourage and affirm their behavior, and help them relieve negative emotions such as shyness, fear, and helplessness.

Environmental Care

Midwives should pay attention to keeping the delivery room clean, spacious, bright, with appropriate temperature and humidity, and strong privacy.

Newborn Care

After the newborn is born, umbilical cord cutting should be delayed, the newborn's respiratory tract cleaned if necessary, the body dried, weighed, footprints made, etc., to provide necessary care and attention to the newborn.

Concerning the Intervention group, the following indicators were investigated.

Labor Pain

During the latent phase (cervix opening to 3 cm), acceleration phase (cervix opening to 7 cm), and deceleration phase (cervix opening to 10 cm), the parturient should be made to point out the value representing the current pain sensation on a 0-10cm ruler (representing 0-10 points). The closer the score is to the total score of 10 points, the stronger the labor pain sensation [6].

Duration of Labor

The average time spent on the first, second, and third stages of labor should be observed and recorded.

Childbirth Attitudes Questionnaire (CAQ)

The CAQ includes four dimensions: self-control, labor pain and injury, and fear of environment and hospital intervention; the total score is 64 points. The closer the score is to the total score of 64 points, the stronger the maternal fear of childbirth [7].

Self-Rating Anxiety Scale (SAS)

The SAS includes 20 items, with a median score of 50 and a total score of 80. The closer the score is to the total score of 80, the stronger the anxiety during childbirth [8].

Delivery Outcome

The use rate of oxytocin, neonatal Apgar score (evaluated at 5 minutes of birth), and 2-hour postpartum bleeding volume should be recorded.

Concerning the Control group (routine nursing intervention), nurses comprehensively monitored and recorded the patient's uterine contractions, fetal heart rate, labor progress, total fetal movement, placental size, among other parameters. After delivery, they moderately massaged the patient's uterus, treated residual membranes and placenta, evaluated the condition of the soft birth canal, and monitored postpartum bleeding.

Statistical analyses

IBM SPSS Statistics for Windows, Version 22 (Released 2013; IBM Corp., Armonk, New York, United States) was used for the statistical analysis. Count data presented as rates (%) and unordered data (oxytocin usage rate) used the c2 test. The measurement data (delivery pain, duration of labor, CAQ, SAS, neonatal Apgar score, and 2-hour postpartum bleeding volume were expressed as mean±standard deviation, and a t-test was performed. P<0.05 was considered statistically significant.

## Results

Patient profiling

**Figure 1:**
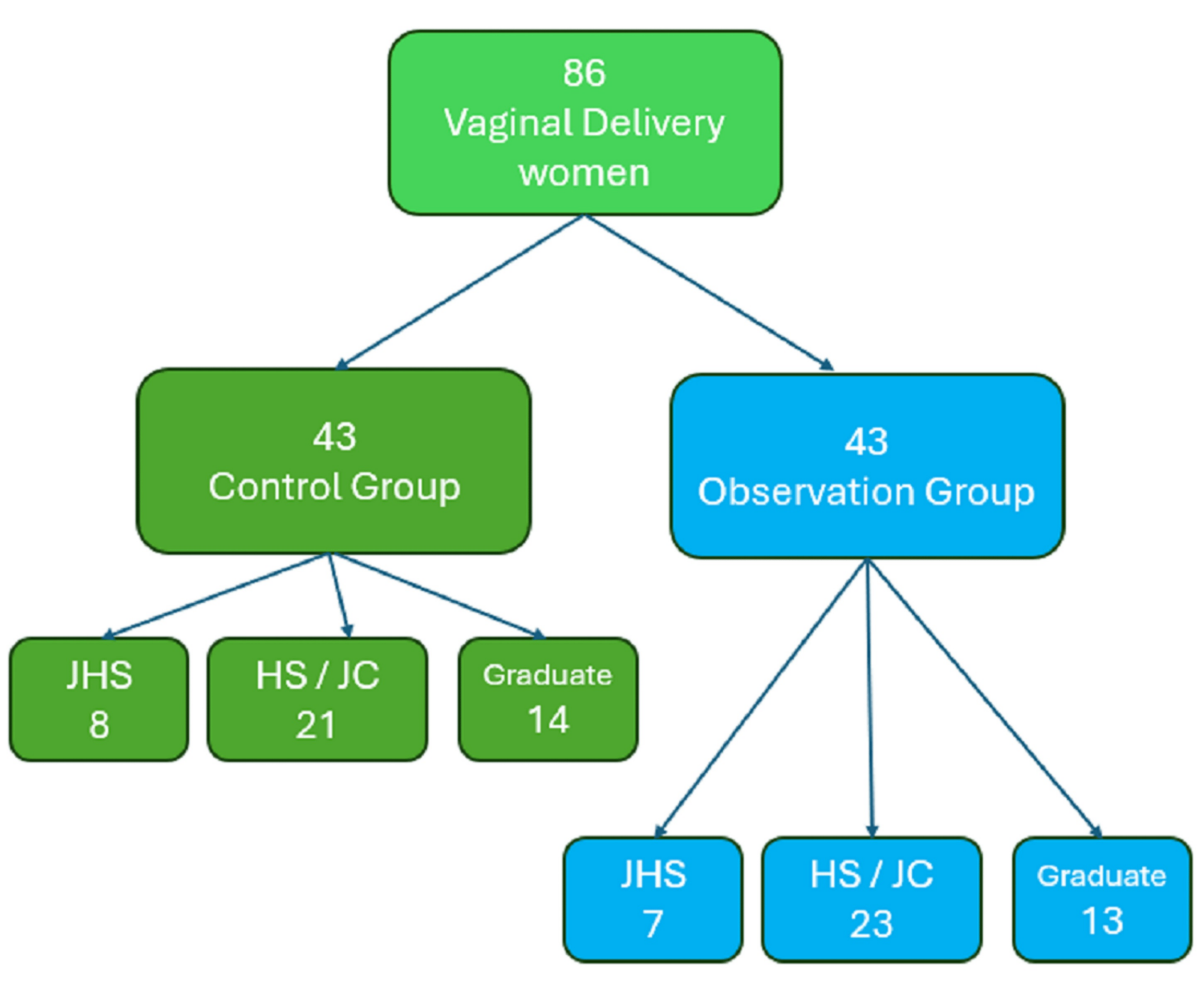
Flowchart depicting patient selection in this study Educational level key: JHS – Junior High School or below; HS / JC – High School / Junior College; Graduate – Bachelor's degree or above

The Intervention group consisted of the following: age: 20-35 (27.92 ± 2.33) years; gestational weeks: 38-42 (39.68±0.29) weeks; weight: 54-76 (64.79± 3.01) Kg, education level: junior high school or below: cases (16.28%), high school or junior college: 23 cases (53.49%), bachelor’s degree or above: 13 cases (30.23%). There was no difference in basic clinical information (such as maternal education level) ( P>0.05) (Figure 1).

The Control group consisted of the following: age: 20-36 (28.18±2.45) years old; gestational weeks: 38-41 (39.48±0.33) weeks; weight: 55-76 (65.10±2.89) Kg; education level: junior high school or below: 8 cases (18.60%), high school or junior college: 21 cases (48.84%), bachelor’s degree or above: 14 cases (32.56%).

Delivery pain

The VAS score of the Intervention group was 3.68, while the Control group in the latent phase was 4.93. The difference between these two means is statistically significant, as indicated by the t-value of 12.169 and a P-value of < 0.001. This suggests that the Intervention group has a significantly lower score in the latent phase compared to the Control group (Table 1).

**Table 1:** Comparison of delivery pain at different time points (latent phase, acceleration phase, deceleration phase) (x±s, P<0.05)

Group	n	Latent phase (scores)	Acceleration phase (scores)	deceleration phase (scores)
Control group	43	4.93±0.51	5.46±0.58	7.93±0.60
Intervention group	43	3.68±0.44	4.87±0.45	7.16±0.49
t-value		12.169	5.270	6.518
P-value		<0.001	<0.001	<0.001

 Duration of labor

The results indicate that, compared to the Control group, the Intervention group exhibited significantly shorter average times in the first, second, and third stages of labor. The data in Table 2 shows that for the first stage of labor, the Control group had an average time of 564.23±45.58 minutes, while the Intervention group had an average time of 433.48±39.87 minutes. For the second stage, the Control group averaged 57.60±5.76 minutes, compared to the Intervention group's 45.32±4.69 minutes. In the third stage, the Control group took 9.64±1.40 minutes, whereas the Intervention group took 6.80±1.13 minutes. Statistical analysis using t-tests revealed highly significant differences (P<0.001) between the two groups in all three stages of labor (P<0.05, shown in Table 2).

**Table 2:** Comparison of duration of labor, depending upon labor stage (x±s, P<0.05)

Group	n	The first stage (min)	The second stage (min)	The third stage (min)
Control group	43	564.23±45.58	57.60±5.76	9.64±1.40
Intervention group	43	433.48±39.87	45.32±4.69	6.80±1.13
t-value		14.158	10.841	10.351
P-value		<0.001	<0.001	<0.001

 Analysis of CAQ and SAS

The results section compares CAQ and SAS scores between a Control group and an Intervention group, each with 43 participants, before and after an intervention. Both groups showed statistically significant decreases in CAQ and SAS scores post-intervention (P<0.001 for all within-group comparisons). Specifically, the control group's CAQ score dropped from 37.89±3.41 to 23.51±2.60, and its SAS score fell from 61.28±1.77 to 47.53±1.40. The intervention group's CAQ score decreased from 38.39±3.22 to 20.37±2.39, while its SAS score went from 60.94±1.68 to 44.81±1.23. Between-group comparisons revealed that the Intervention group had a more significant improvement in both CAQ and SAS scores than the Control group, with t-values of 5.830 and 9.571, respectively, both statistically significant at P<0.001 (Table 3).

**Table 3:** Comparison of CAQ and SAS survey scores pre-/post-intervention (x±s, P<0.05) Note: Comparison before and after intervention within the group, P<0.05.

Group	n	CAQ (scores)		SAS (scores)	
		Before intervention	After intervention	Before intervention	After intervention
Control group	43	37.89±3.41	23.51±2.60^a^	61.28±1.77	47.53±1.40^a^
Intervention group	43	38.39±3.22	20.37±2.39^a^	60.94±1.68	44.81±1.23^a^
t-value		0.699	5.830	0.914	9.571
P-value		0.486	<0.001	0.364	<0.001

Outcome of childbirth

Compared with the control group, the intervention group had a lower usage rate of oxytocin and reduced two-hour postpartum bleeding volume (P<0.05). There was no difference in Apgar scores at 5 minutes of birth for newborns between groups (P<0.05) (Table 4).

**Table 4:** Comparison of outcome of childbirth, according to percentage usage rate of oxytocin, newborn Apgar score and 2-hour postpartum bleeding volume readings (x±s, P<0.05)

Group	n	Usage rate of oxytocin (%)	Apgar scores of newborns (scores)	2-hour postpartum bleeding volume (mL)
Control group	43	21（48.84）	9.25±0.21	198.45±16.74
Intervention group	43	6（13.95）	9.30±0.22	165.83±13.92
c^2^/t-value		12.147	1.078	9.825
P-value		<0.001	0.284	<0.001

## Discussion

Overall, this study showed that the VAS scores of the Intervention group at different time points (latent phase, acceleration phase, deceleration phase) were lower (P<0.05), the Intervention group had a shorter delivery time for all stages of labor (P<0.05), together with lower scores of CAQ and SAS (P<0.05), and had a lower usage rate of oxytocin and less 2-hour postpartum bleeding volume (P<0.05).

Childbirth is a natural phenomenon of reproducing offspring, during which the mother's psychology and body undergo significant changes. Especially during vaginal delivery, there is often intense uterine contractions and pain, which can cause anxiety, fear, and tension in vaginal delivery women. This can lead to a lack of confidence in vaginal delivery and affect the progress and outcome of the labor process. Therefore, it is necessary to strengthen the nursing intervention [9-13].

This study showed that the VAS scores of the Intervention group at different time points (latent phase, acceleration phase, deceleration phase) were lower (P<0.05). Nursing intervention during childbirth can alleviate the pain caused by uterine contractions at different time points by guiding vaginal delivery women to adjust their breathing and master relaxation techniques, combined with deep breathing, massage, comfortable positions, and other methods. At the same time, by providing psychological support, encouraging vaginal delivery women to express their feelings, and appropriate massage, it can also help them relieve tension and pain. In addition, closely observing the situation of vaginal delivery women and taking corresponding measures according to the specific situation, such as adjusting positions and using painkillers, can also alleviate delivery pain [14-16].

This study showed that the Intervention group had shorter delivery times for all stages of labor (P<0.05). Nursing intervention during childbirth involved closely observing and recording key indicators, such as the mother's vital signs, frequency and intensity of contractions, and fetal heart rate, during the delivery process. This enabled rapid and accurate handling of various delivery situations, ensuring smooth delivery and avoiding delays [17,18]. Through psychological intervention, encouragement, and support, it can help vaginal delivery women alleviate anxiety and fear, enhance confidence, maintain a good psychological state, and contribute to the smooth progress of the labor process [19]. Providing reasonable guidance based on different stages of the labor process, such as adjusting breathing and changing positions, can optimize fetal descent and the delivery process, alleviate maternal pain and discomfort, and ensure smooth progress of the labor process. Therefore, strengthening nursing intervention during childbirth can shorten the various stages of labor for women undergoing vaginal delivery.

This study showed that the Intervention group had lower scores of CAQ and SAS (P<0.05). Nursing intervention during childbirth provided comprehensive explanations of the vaginal delivery process to the mother, which can help her understand and overcome her fear of the unknown. At the same time, attention should be paid to listening to the mother's feelings, physical and mental needs, and environmental needs, and appropriate advice and comfort should be given. This can make the mother feel cared for and supported [20,21]. By using diversified pain relief methods, such as adjusting breathing and massage, it is possible to effectively alleviate delivery pain and reduce anxiety and fear. Providing a quiet, comfortable, and humane delivery environment also helped mothers relax their bodies and minds and relieve tension. Therefore, strengthening nursing intervention during childbirth can alleviate the fear and anxiety of vaginal delivery mothers.

This study showed that the Intervention group had a lower usage rate of oxytocin and less 2-hour postpartum bleeding volume (P<0.05). Nursing interventions during childbirth can alleviate the tension of vaginal delivery women by providing psychological support, guiding them in correct breathing and relaxation techniques, and reducing the pressure during delivery, thereby reducing the demand for oxytocin. At the same time, early observation and recording of vaginal bleeding after delivery, timely massage of the uterus, etc. can promote uterine contractions and reduce postpartum bleeding [22,23].

Study limitations

Notwithstanding all efforts placed in this research endeavor, this study does have limitations. They include the limited cohort size of <50 participants in each group since this study was carried out within a single-center setting, and thus was dependent upon admission rate during the fixed study period (July 2023 to August 2024) The use of a single-center setting could also have limited the generalizability of the findings to other settings. Furthermore, possible bias could have been introduced during patient selection due to the evaluation of the educational level of the mothers participating in this study. In addition, the data obtained from the CAQ and SAS score surveys could also be biased, as it was subjective to the current emotional state of the individual study participant during such a momentous life event, that is, child delivery. Consequently, the study design likely did not involve blinding, which could introduce bias, particularly in the assessment of subjective outcomes such as pain and anxiety. The study also primarily focused on immediate effects during labor and the early postpartum period. It did not assess the long-term impact of the intervention on maternal well-being or postpartum recovery. Future research on this study can include a cost-effectiveness study for the actual routine implementation of such augmented nursing interventions within a hospital setting, particularly concerning the costs involved for any specialized staff training and/or recruitment possibilities.

## Conclusions

This study emphasizes the vital role of enhanced nursing interventions during vaginal delivery in improving maternal experiences and birth outcomes. Providing effective nursing care can significantly reduce labor pain, support the natural progression of childbirth, and ease maternal anxiety and fear, ultimately contributing to better overall delivery results. By integrating evidence-based nursing approaches, healthcare professionals can foster a more comfortable and reassuring birthing environment, benefiting both mothers and newborns. A key finding of this study is that targeted nursing interventions help alleviate pain through techniques such as breathing exercises, guided relaxation, and strategic positioning. These methods not only enhance comfort but also empower women by encouraging active participation in labor, fostering confidence and control over the birthing process.
